# Supplementary material for: Generation of human iPSC-derived phrenic-like motor neurons to model respiratory motor neuron degeneration in ALS
Source: Commun Biol. 2024 Feb 28;7:238. doi: 10.1038/s42003-024-05925-z (PMC10901792; doi:10.1038/s42003-024-05925-z)
Supplement: Supplementary file 2 — Supplementary Information [file 42003_2024_5925_MOESM2_ESM.pdf]

Supplementary Figures

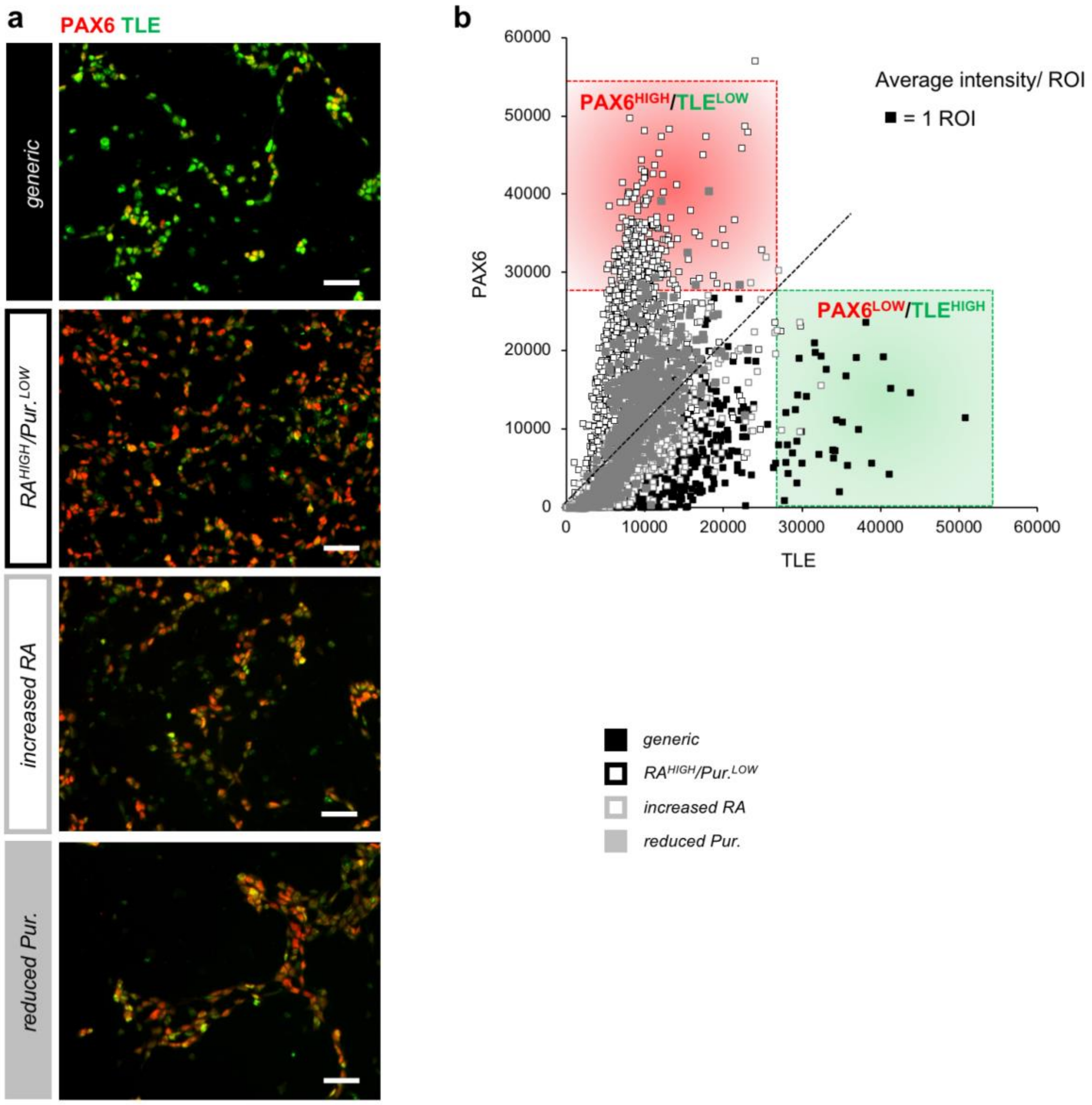

Supplementary Figure 1. Identification of PAX6^{HIGH}/TLE^{LOW} MNP cells. (a) Representative images of MNP cultures stained with anti-PAX6 (red) and anti-TLE (green) antibodies after 12 days of differentiation under each of the four experimental conditions defined in Figure 1a. Scale bars = 50 μm. **(b)** Plot of the fluorescence intensity of PAX6 and TLE staining for each cell (e.g., region of interest (ROI)) present in each representative image presented in (a). Cells that are PAX6^{LOW}/TLE^{HIGH} are represented in the green rectangular area and correspond mostly to cells of the *generic* condition (black squares). In contrast, cells that are PAX6^{HIGH}/TLE^{LOW} are represented in the red rectangular area and correspond mostly to cells of the *RA^{HIGH}/Pur.^{LOW}* condition (hollow black squares).

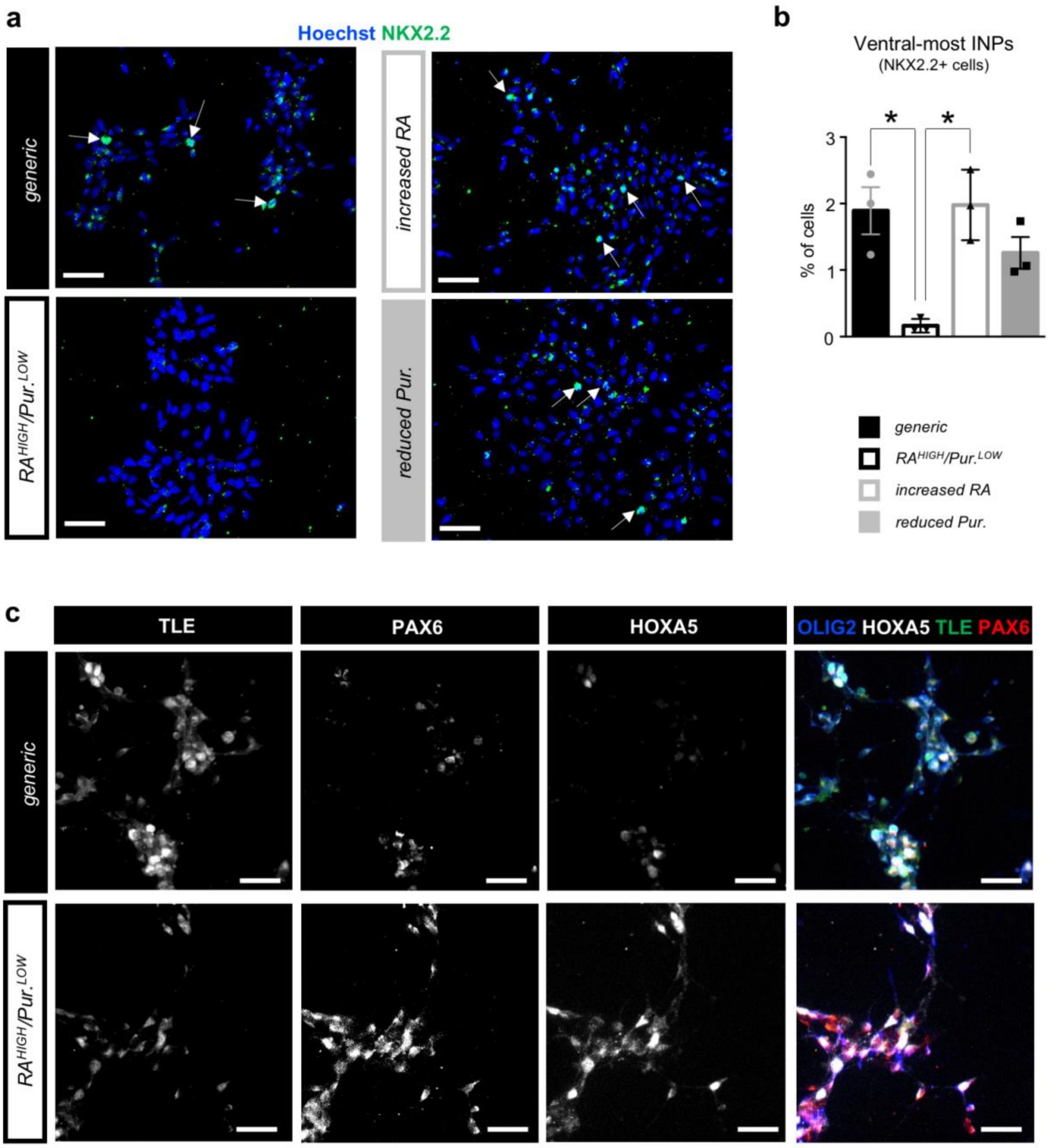

Supplementary Figure 2. Dorsalization and caudalization of motor neuron progenitor cultures. (a) Representative images of MNP cultures stained with the anti-NKX2.2 antibody after 12 days of differentiation in each of the four experimental conditions defined in Figure 1A. **(b)** Quantification of the proportion of ventral-most interneuron progenitors identified as NKX2.2+ cells. Friedman non parametric test and Dunn's post-hoc multiple comparisons test; *=$p<0.05$; N = 3 biologically independent cultures (with >500 cells in random fields for each culture). Error bars are means ± standard error of means (SEM) of the average. **(c)** Representative images of MNP cultures stained with anti-HOXA5 (white), anti-PAX6 (red), anti-TLE (green) and anti-OLIG2 (blue) antibodies after 12 days of differentiation in the *generic* and the *RA*^{HIGH}*/Pur.*^{LOW} conditions. Scale bars = 50 µm.

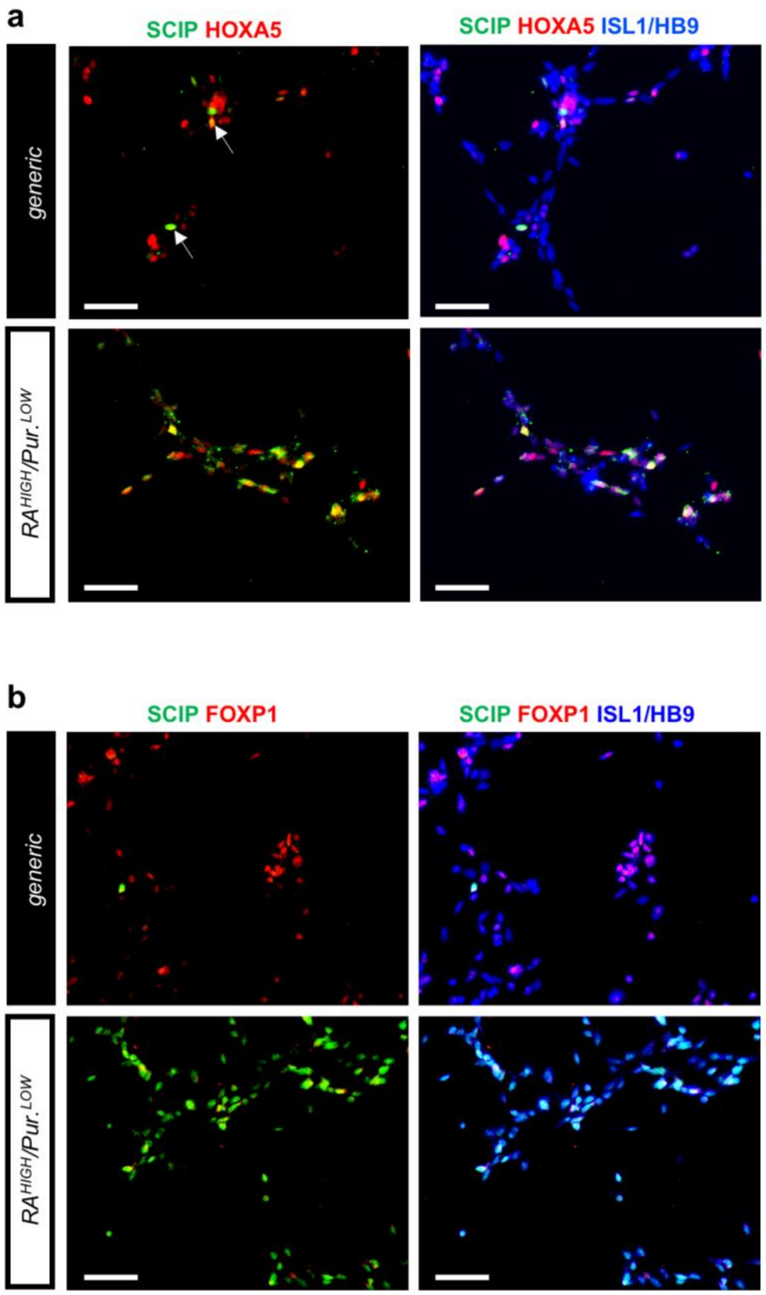

Supplementary Figure 3. SCIP^{+} phrenic motor neurons express HOXA5 but not FOXP1. **(a)** Representative images of MNs co-stained with pan-MN marker HB9/ISL1 (blue), anti-HOXA5 (red), and anti-SCIP (green) antibodies after 25 days of culture in the *generic* and the *RA^{HIGH}/Pur.^{LOW}* conditions. **(b)** Representative images of MNs co-stained with anti-HB9/ISL1 (blue), anti-FOXP1 (red), and anti-SCIP (green) antibodies after 25 days of culture in the *generic* and the *RA^{HIGH}/Pur.^{LOW}* conditions. Scale bars = 50 µm.

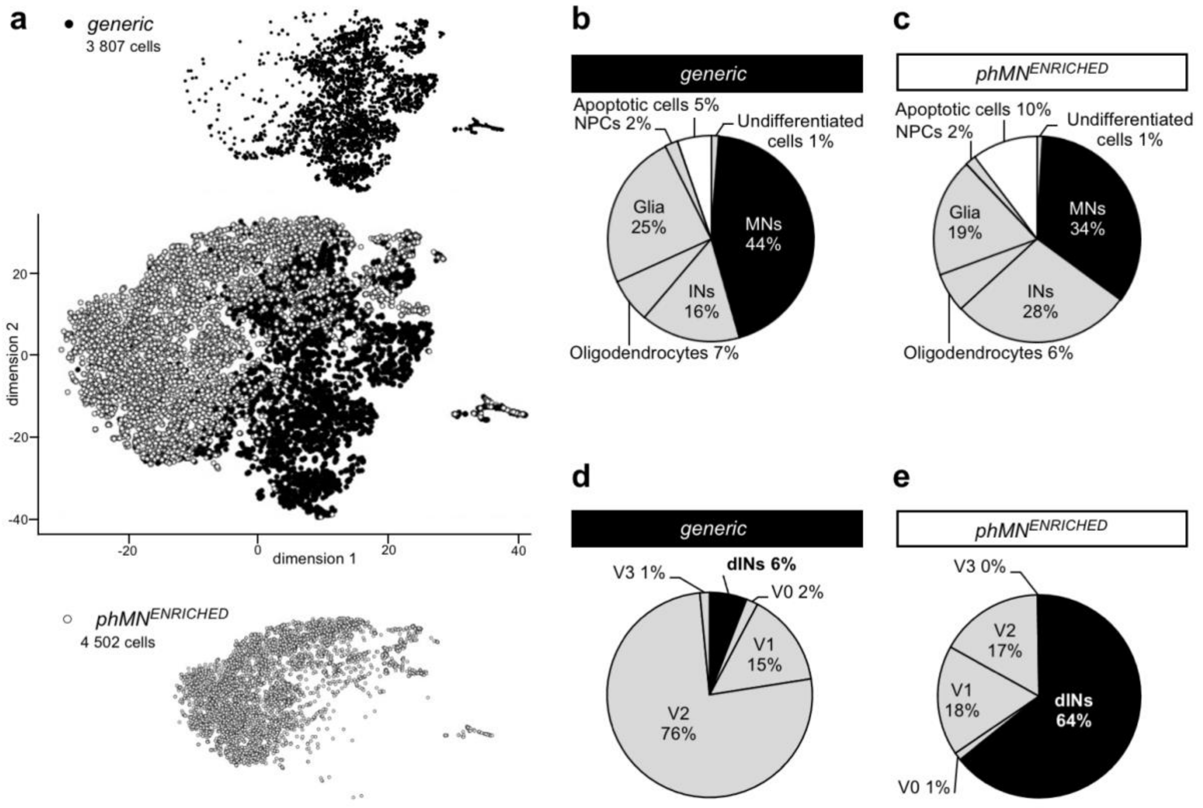

Supplementary Figure 4. Heterogenous composition of human iPSC-derived motor neuron culture defined by single-cell RNA sequencing. (a) t-SNE plot of hiPSC derived MN cultures after 28 days of differentiation in the *generic* (black dots) or the *phMN^{ENRICHED}* (white dots) condition. **(b-c)** Relative proportion of undifferentiated cells, NPCs, INs, MNs, astrocytic glial cells, oligodendrocytes, and apoptotic cells, identified after 28 days of differentiation in the *generic* condition **(b)** or with *phMN^{ENRICHED}* **(c)**, based on the specific expression of the following genes: *NANOG* and *OCT4* for undifferentiated cells; *SOX1*, *SOX2*, and *MKI67* for NPCs; *PAX2*, *PAX3*, *LBX1*, *EVX1*, *EN1*, *CHX10*, *GATA3*, *SOX14*, *SIM1*, *TLX3* for INs; *NEUROG2*, *OLIG2*, *HB9*, *ISL1*, *ISL2*, *CHAT* for MNs; *S100B and SOX9* for astrocytic glial cells; *PDGFRα* and *GALC* for oligodendrocytes; *BCL2*, *BAX*, and *NGF* for apoptotic cells. For both conditions, the proportion of MNs is represented in black. **(d-e)** Proportion of each of the spinal interneuron (IN) subtypes, identified after 28 days of differentiation in the *generic* **(d)** or the *phMN^{ENRICHED}* **(e)** condition, based on combinatorial gene expression: dorsal INs (dINs), *LBX1^{+}/PAX2^{+}/TLX3^{+}*; V0 INs, *EVX1^{+}/EN1^{-}*; V1 INs, *EVX1^{-}/EN1^{+}*; V2 INs, *CHX10^{+}/SOX14^{+}/GATA3^{+}*; V3 INs, *SIM1^{+}/NKX2.2^{+}*. For both conditions, the proportion of dINs is represented in black.

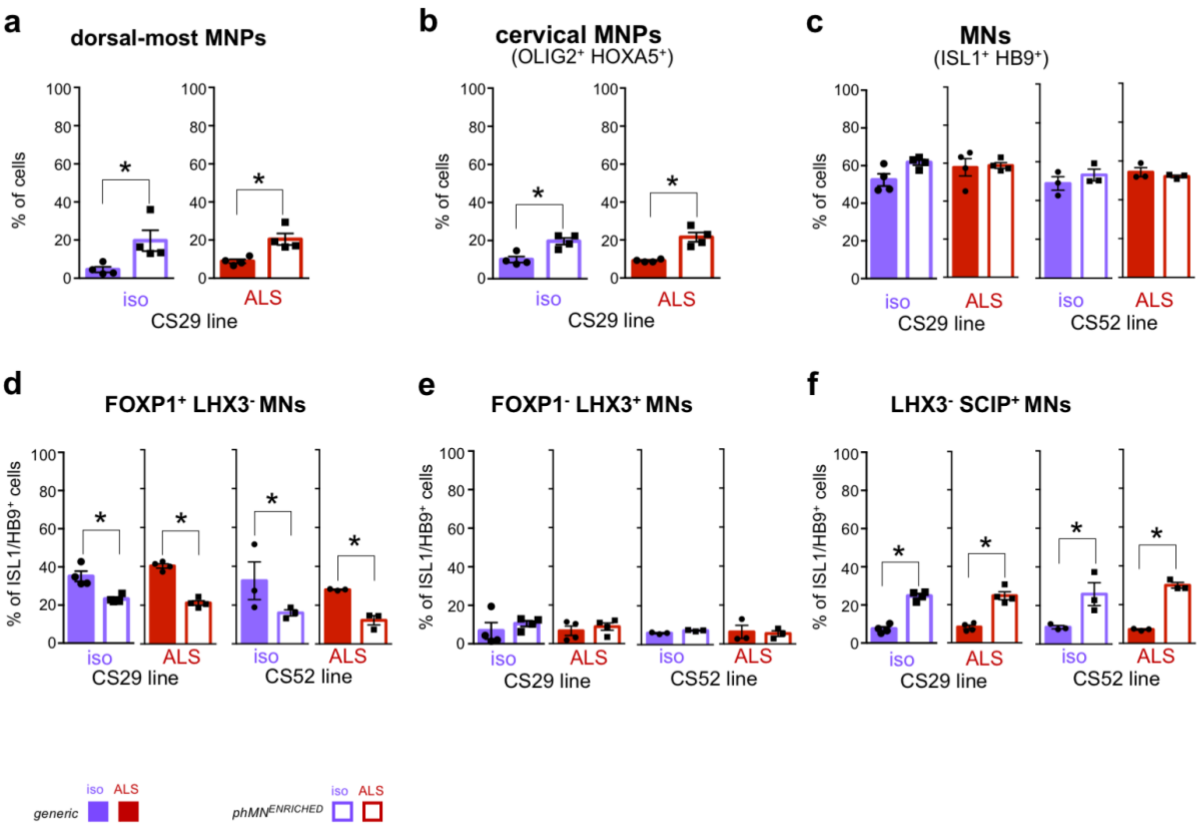

Supplementary Figure 5. Enrichment in dorsal-most cervical motor neuron progenitors and phrenic motor neurons derived from two *C9orf72* iPSC lines. (a-b) Quantification of the dorsal-most MNPCs (e.g., PAX6^{high}/TLE^{low} OLIG2^{+} cells, (**a**) and the cervical MNPCs (e.g., HOXA5^{+}/OLIG2^{+} cells, (**b**) as percentages of the total number of cells after 12 days of differentiation in either the *generic* or the *phMN^{ENRICHED}* culture conditions established in previous figures. **(c)** Quantification of MNs (e.g., ISL1^{+}/HB9^{+} cells) as percentages of the total number of cells after 25 days of differentiation. **(d-f)** Quantification of the LMC MNs identified as FOXP1^{+}/LHX3^{-} **(d)**, MMC MNs identified as LHX3^{+}/FOXP1^{-} **(e)**, and phMNs identified as LHX3^{-}/SCIP^{+} **(f)** as percentages of the number of ISL1^{+}/HB9^{+} cells. MMC = median motor column, LMC = lateral motor column, phMNs = phrenic MNs. Mann-Whitney non parametric test; *=$p<0.05$; N= 4 biologically independent cultures for CS29 and N=3 biologically independent cultures for CS52 (with >500 cells in random fields for each culture). Error bars are means ± standard error of means (SEM) of the average.

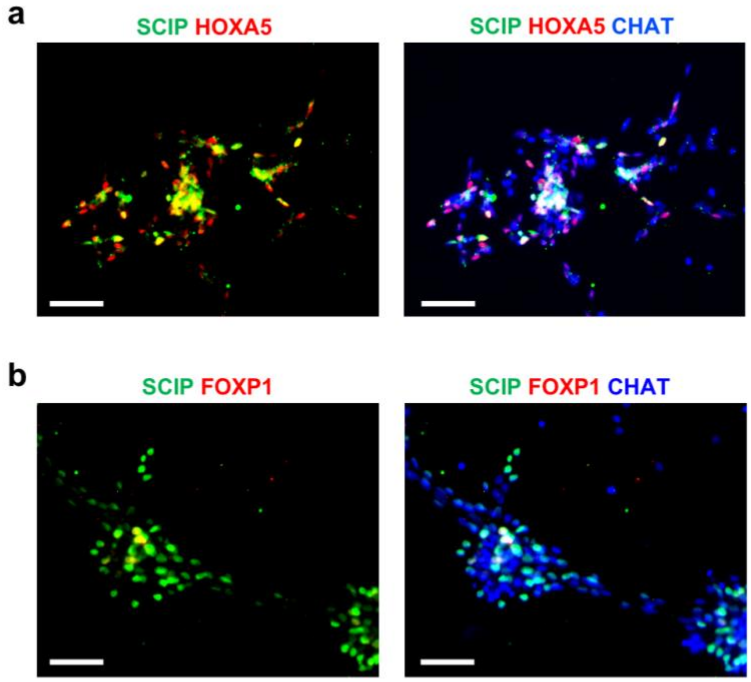

Supplementary Figure 6. Post-FACS SCIP$^+$ phrenic motor neurons express HOXA5 but not FOXP1. Representative images of MNs co-stained with anti-CHAT (blue), anti-SCIP (green) antibodies, and either anti-HOXA5 antibody (red, **a**) or anti-FOXP1 antibody (red, **b**), two-weeks after FACS sorting of phMN^{ENRICHED} cultures from ALS CS52 iPSCs (D32). Scale bars = 50 µm.

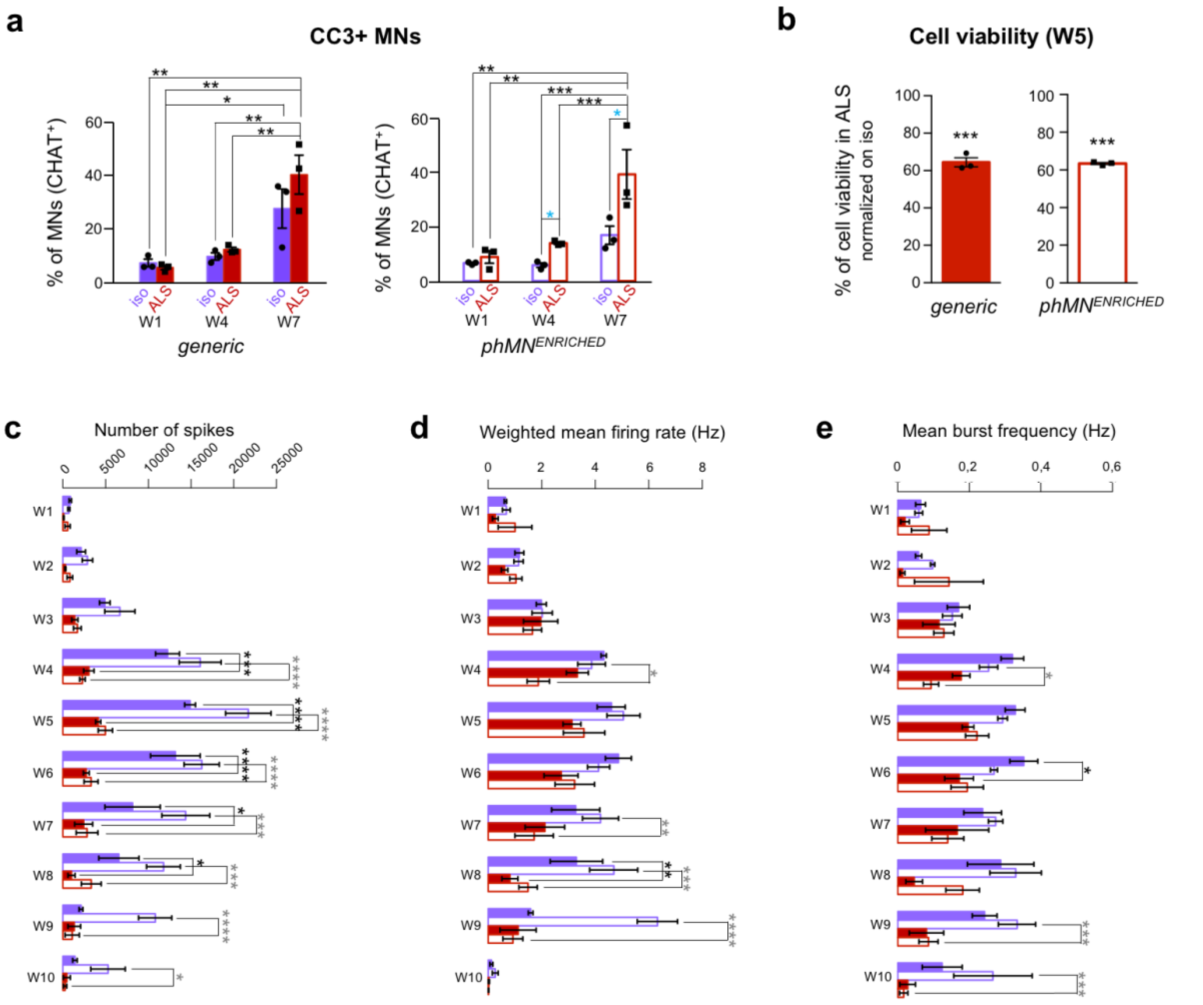

Supplementary Figure 7. *C9orf72* ALS motor neuron death in 'generic' or 'enriched' cultures derived from the CS52 hiPSC line. (a) Quantification of $CC3^{+}$ MNs (e.g., $CC3^{+}$/$CHAT^{+}$ cells) as percentages of the total number of MNs (e.g., $CHAT^{+}$ cells) in *generic* (left, filled bars) or *phMN*^{ENRICHED} (right, hollow bars) cultures derived from the CS52 hiPSC line. Two-way ANOVA and Tukey's post-hoc multiple comparisons test; *=$p<0.05$; **=$p<0.005$; ***=$p<0.0005$; N = 3 biologically independent cultures per condition. Blue asterisks show statistical significance between Iso and ALS at the same time point. **(b)** Cell viability assay measured using CellTiter-GLO after 60 days of differentiation (i.e., five-weeks (W5) post-plating) in *generic* (left, filled bars) or *phMN*^{ENRICHED} (right, hollow bars) cultures derived from the CS52 iPSC line. Cell viability for ALS cells was normalized on each respective corresponding isogenic control. Wilcoxon signed rank test; ***=$p<0.0001$; N = 3 biologically independent cultures per condition. **(c-e)** Histograms showing the average number of spikes (**c**)**,** weighted mean firing rate (Hz) (**d**), and mean burst frequency (Hz) (**e**) over time, from one-week to 10-weeks post-plating of isogenic *generic*, isogenic *phMN*^{ENRICHED}, ALS *generic*, and ALS *phMN*^{ENRICHED} from the CS52 hiPSC line. Two-way repeated measure

ANOVA and Tukey's post-hoc multiple comparisons test; *=p<0.05; **=p<0.005; ***=p<0.0005; ****=p<0.0001; N = 3 biologically independent cultures per condition. Black asterisks = statistical significance for isogenic vs ALS *generic* cultures; Grey asterisks = statistical significance for isogenic vs ALS *phMN*^{ENRICHED} cultures. Error bars are means ± standard error of means (SEM) of the average.

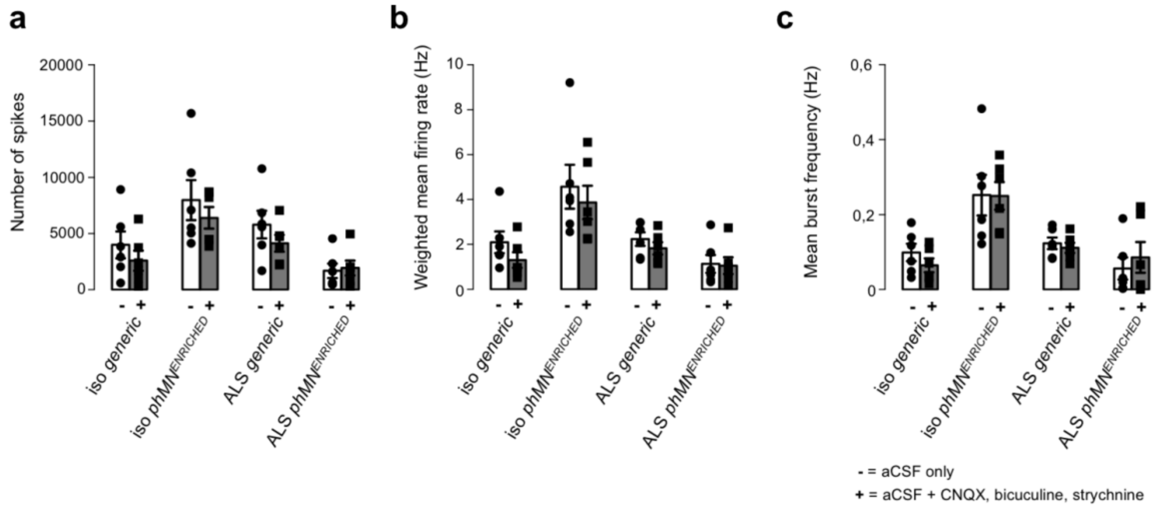

Supplementary Figure 8. Histograms showing the average number of spikes (**a**)**,** weighted mean firing rate (Hz) (**b**), and mean burst frequency (Hz) (**c**) at 5-weeks (D60) post-plating for each of the four culture conditions derived from the CS29 line in the absence (white bars; '-') or presence (grey bars; '+') of drugs suppressing all synaptic currents (CNQX, bicuculine and strychnine). Mann-Whitney non-parametric test comparing data in the absence or presence of drugs; no statistically significant difference; N = 6 biologically independent cultures per condition. Error bars are means ± standard error of means (SEM) of the average.
