# Supplementary material for: Generation of human iPSC-derived phrenic-like motor neurons to model respiratory motor neuron degeneration in ALS
Source: Commun Biol. 2024 Feb 28;7:238. doi: 10.1038/s42003-024-05925-z (PMC10901792; doi:10.1038/s42003-024-05925-z)
Supplement: Supplementary file 3 — Reporting Summary [file 42003_2024_5925_MOESM3_ESM.pdf]

nature portfolio

Corresponding author(s): COMMSBIO-23-2625-T

Last updated by author(s): Jan 9, 2024

Reporting Summary

Nature Portfolio wishes to improve the reproducibility of the work that we publish. This form provides structure for consistency and transparency in reporting. For further information on Nature Portfolio policies, see our Editorial Policies and the Editorial Policy Checklist.

Statistics

For all statistical analyses, confirm that the following items are present in the figure legend, table legend, main text, or Methods section.

n/a	Confirmed	
☐	☒	The exact sample size (*n*) for each experimental group/condition, given as a discrete number and unit of measurement
☐	☒	A statement on whether measurements were taken from distinct samples or whether the same sample was measured repeatedly
☐	☒	The statistical test(s) used AND whether they are one- or two-sided<br>*Only common tests should be described solely by name; describe more complex techniques in the Methods section.*
☒	☐	A description of all covariates tested
☐	☒	A description of any assumptions or corrections, such as tests of normality and adjustment for multiple comparisons
☐	☒	A full description of the statistical parameters including central tendency (e.g. means) or other basic estimates (e.g. regression coefficient) AND variation (e.g. standard deviation) or associated estimates of uncertainty (e.g. confidence intervals)
☒	☐	For null hypothesis testing, the test statistic (e.g. *F*, *t*, *r*) with confidence intervals, effect sizes, degrees of freedom and *P* value noted<br>*Give P values as exact values whenever suitable.*
☒	☐	For Bayesian analysis, information on the choice of priors and Markov chain Monte Carlo settings
☒	☐	For hierarchical and complex designs, identification of the appropriate level for tests and full reporting of outcomes
☒	☐	Estimates of effect sizes (e.g. Cohen's *d*, Pearson's *r*), indicating how they were calculated

Our web collection on statistics for biologists contains articles on many of the points above.

Software and code

Policy information about availability of computer code

Data collection	The raw sc-RNAseq data (FASTQ files) were first processed using the Cell Ranger pipeline (10x Genomics) at the IRIC's Bioinformatics Platform (https://bioinfo.iric.ca/).
Data analysis	Post Cell Ranger data were imported into R and all the subsequent data analysis was conducted in R version 3.5.2 using Bioconductor Software Version 3.8 (http://bioconductor.org). The R workflow used for the analysis was adapted from a published workflow used to characterize hiPSC-derived MN cultures (Thiry et al. 2020), available publicly on GitHub (https://github.com/regan-hamel/h-iPSCs-MNI).

For manuscripts utilizing custom algorithms or software that are central to the research but not yet described in published literature, software must be made available to editors and reviewers. We strongly encourage code deposition in a community repository (e.g. GitHub). See the Nature Portfolio guidelines for submitting code & software for further information.

Data

Policy information about availability of data

All manuscripts must include a data availability statement. This statement should provide the following information, where applicable:

- Accession codes, unique identifiers, or web links for publicly available datasets
- A description of any restrictions on data availability
- For clinical datasets or third party data, please ensure that the statement adheres to our policy

The filtered sc-RNAseq data used in the present study will be available on GitHub (https://github.com/LouiseThi/scRNAseq-hiPSC-phrenic-MNs).

Human research participants

Policy information about studies involving human research participants and Sex and Gender in Research.

Reporting on sex and gender	NA
Population characteristics	NA
Recruitment	NA
Ethics oversight	NA

Note that full information on the approval of the study protocol must also be provided in the manuscript.

Field-specific reporting

Please select the one below that is the best fit for your research. If you are not sure, read the appropriate sections before making your selection.

☒ Life sciences ☐ Behavioural & social sciences ☐ Ecological, evolutionary & environmental sciences

For a reference copy of the document with all sections, see nature.com/documents/nr-reporting-summary-flat.pdf

Life sciences study design

All studies must disclose on these points even when the disclosure is negative.

Sample size	Five human iPSC lines were used: the NCRM-1 control line, the ALS-patient derived line CS29iALS-C9nxx and its matching isogenic control CS29iALS-C9n1.ISOnxx, the ALS-patient derived line CS52iALS-C9nxx and its matching isogenic control CS52iALS-C9n6.ISOnxx.
Data exclusions	For immunocytochemistry characterization, no data were excluded from the study.<br>For single-cell RNA sequencing experiments, cells that did not meet quality control were likely damaged cells and were therefore excluded from the study.<br>Following FACS-based cell sorting, no data were excluded from the study.<br>For cell death assay, no data were excluded from the study.<br>For MEA experiments, all probes were visualized immediately before each recording session to confirm a full monolayer of cells: plates were groups of cells had detached from the well were excluded from the study.
Replication	For each experiment and each experimental group, cells were generated from each cell line at least 3 separate times.<br>For single-cell RNA sequencing, the experiment was not repeated because of the high cost. However, unpublished data obtained from more mature samples confirm the experimental findings described in this study.<br>For MEA, experiments were repeated up to 10 times to account for variability inherent to the method.
Randomization	For each cell line and each experimental group, all experiments were replicated at least 3 times.
Blinding	Investigators were blinded to group allocation during data collection and analysis

Reporting for specific materials, systems and methods

We require information from authors about some types of materials, experimental systems and methods used in many studies. Here, indicate whether each material, system or method listed is relevant to your study. If you are not sure if a list item applies to your research, read the appropriate section before selecting a response.

Materials & experimental systems

n/a	Involved in the study
☐	☒ Antibodies
☐	☒ Eukaryotic cell lines
☒	☐ Palaeontology and archaeology
☒	☐ Animals and other organisms
☒	☐ Clinical data
☒	☐ Dual use research of concern

Methods

n/a	Involved in the study
☒	☐ ChIP-seq
☒	☐ Flow cytometry
☒	☐ MRI-based neuroimaging

Antibodies

Antibodies used	mouse anti-OLIG2 (1/100; Millipore Corp.; Billerica, MA, USA; Cat. No. MABN50);<br>goat anti-OLIG2 (1/200; R&D Systems; Cat. No. AF2418);<br>rabbit anti-PAX6 (1/500; Covance; Emeryville, CA, USA; Cat. No. PRB-278P);<br>mouse anti-PAX6 (1/150; Millipore Corp.; Cat. No. MAB5554);<br>rat panTLE antibody (1/10) (Marçal, N. et al. Mol Cell Biol, 25 (2005));<br>rabbit anti-HOMEOBOX A5 (HOXA5) (1/150; kindly provided by Dr. Jeremy Dasen, New York University School of Medicine, New York, NY);<br>mouse anti-NK2 HOMEOBOX 2 (NKX2.2) (1/100; DSHB; Cat. No. 74.5A5-c);<br>mouse anti-HOMEOBOX PROTEIN HB9 (HB9) (1/30; DSHB; Cat. No. 81.5C10-c);<br>mouse anti-ISLET1 (ISL1) (1/30; DSHB; Cat. No. 39.4D5-c);<br>rabbit anti-LIMB HOMEOBOX CONTAINING 3 (LHX3) (1/100; Abcam; Toronto, ON, Canada; Cat. No. ab14555);<br>goat anti-FORKHEAD BOX PROTEIN 1 (FOXP1) (1/100; R&D Systems; Cat. No. AF4534);<br>mouse anti-neurofilament protein (2H3) (1/35; DSHB; Cat. No. 2H3-c);<br>goat anti-CHAT (1/100; Millipore; Cat. No. MAB144P);<br>guinea pig anti-SCIP (1/16,000; kindly provided by Dr. Jeremy Dasen).
Validation	The rat panTLE antibody was validated by previous groups (Marçal, N. et al. Mol Cell Biol, 25 (2005));<br>The rabbit anti-HOXA5 antibody was validated by previous groups (Dasen et al. Cell, 2005; Philippidou et al. Nat Neurosci., 2012);<br>The guinea pig anti-SCIP antibody was validated by previous groups (Dasen et al. Cell, 2005; Philippidou et al. Nat Neurosci., 2012);<br>All other primary antibodies used in this study were validated by the manufacturers and previous groups. Certificates of analysis are publicly available on the manufacturer's web page.<br>mouse anti-OLIG2: Immunocytochemistry Analysis: This antibody has been shown to detect Olig2, as reported by an independent laboratory (Lee, S.K., et al. (1995). Genes & Dev. 19:282-294).<br>goat anti-OLIG2: Salazar DL et al., PLoS ONE, 2010-08-18;5(8):e12272; Piltti K et al., Stem Cells Transl Med, 2013-02-14;2(3):204-16.<br>rabbit anti-PAX6: Zhang Y, et al., Dev Biol. 2014 Dec 1;396(1):19-30<br>mouse anti PAX6: Engelkamp, D, et al. Development, 126: 3585-96 (1999) 1999<br>mouse anti-NKX2.2: 29 references; Initial publication: Ericson et al., Cell 90.1 (1997 Jul 11): 169-80.<br>mouse anti-HB9: 54 references; Initial publication: Tanabe et al., Cell 95.1 (1998 Oct 2): 67-80<br>mouse anti-ISLET1: 62 references; Initial publication: Tsuchida et al., Cell 79.6 (1994 Dec 16): 957-70.<br>rabbit anti-LHX3: 25 references; 4 references for ICC: Da Costa et al., Frontiers in Cell and Developmental Biology, vol. 10, 2022.<br>goat anti-FOXP1: 7 references; certificates of analysis are publicly available.<br>mouse anti-neurofilament protein (2H3): 100 references; Initial reference: Dodd et al., Neuron 1.2 (1988 Apr): 105-16<br>goat anti-CHAT: More than 100 references; certificates of analysis are publicly available.

Eukaryotic cell lines

Policy information about cell lines and Sex and Gender in Research

Cell line source(s)	The human NCRM-1 line was obtained from the National Institutes of Health Stem Cell Resource (Bethesda, MD, USA).<br>The ALS-patient derived lines and their matching isogenic controls were obtained from Cedars-Sinai (Los Angeles, CA, USA).
Authentication	Each cell line was authentified by the providing entity.
Mycoplasma contamination	All cultures tested negative for mycoplasma contamination
Commonly misidentified lines<br>(See ICLAC register)	No commonly misidentified line was used in this study
